# Supplementary material for: Removal of Erythromycin from Water by Ibuprofen-Driven Pre-Organized Divinyl Sulfone Cross-Linked Dextrin
Source: Polymers (Basel). 2024 Apr 13;16(8):1090. doi: 10.3390/polym16081090 (PMC11055069; doi:10.3390/polym16081090)
Supplement: Supplementary file 1 [file polymers-16-01090-s001.zip › polymers-2920934-supplementary.pdf]

Supplementary Materials

Removal of Erythromycin from Water by Ibuprofen-Driven Pre-Organized Divinyl Sulfone Cross-Linked Dextrin

Mariano Ortega-Muñoz [1,2,3], Sarah Alvarado [1,†], Alicia Megia-Fernandez [1,2,3], Fernando Hernandez-Mateo [1,2,3], Francisco Javier Lopez-Jaramillo [1,2,3,*] and Francisco Santoyo-Gonzalez [1,2,3,*]

1 Department of Organic Chemistry, Faculty of Sciences, University of Granada, 18073 Granada, Spain
2 Unit of Excellence in Chemistry Applied to Biomedicine and the Environment, University of Granada, 18073 Granada, Spain
3 Biotechnology Institute, University of Granada, 18071 Granada, Spain
* Correspondence: fjljara@ugr.es (F.J.L.-J.); fsantoyo@ugr.es (F.S.-G.)
† Current address: Institute for Advanced Chemistry of Catalonia, CSIC, 08034 Barcelona, Spain.

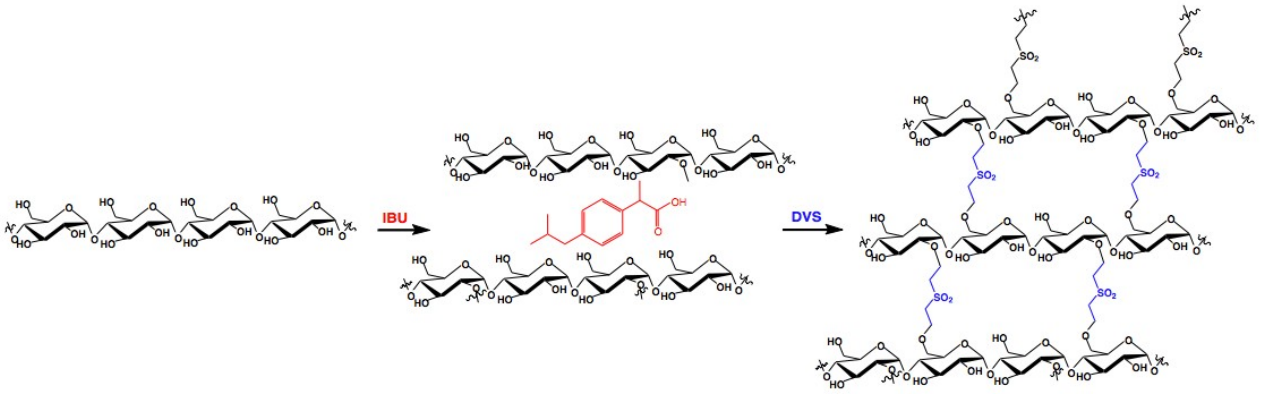

Scheme S1. Synthesis of the ibuprofen (IBU) driven preorganized divinyl sulfone (DVS) cross-linked dextrin

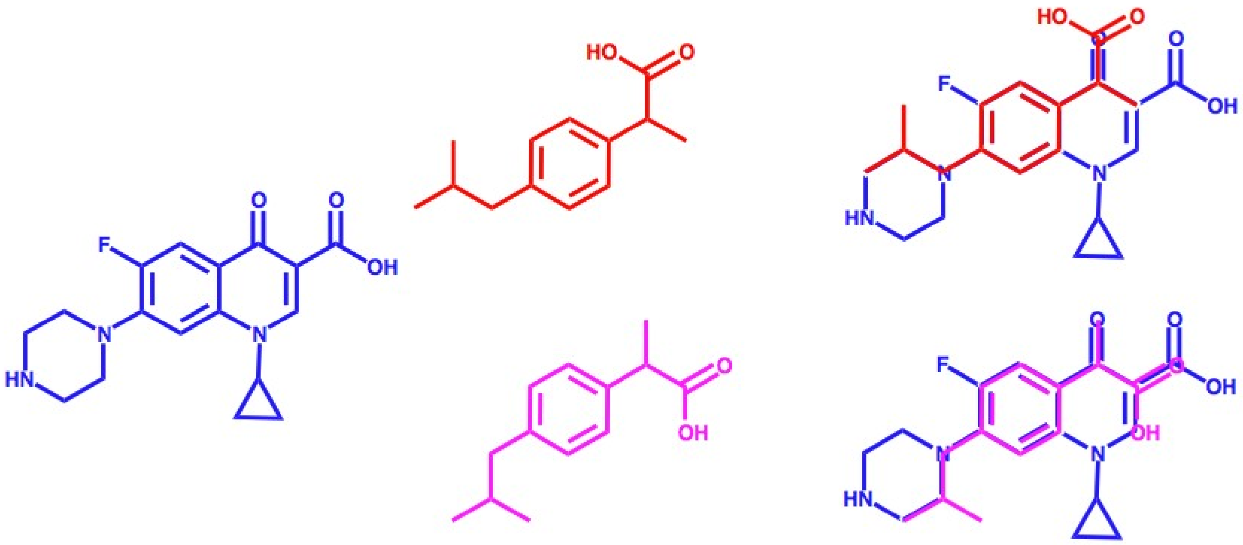

Figure S1. Superimposition of two conformers of ibuprofen (red and magenta) on ciprofloxacin (blue).

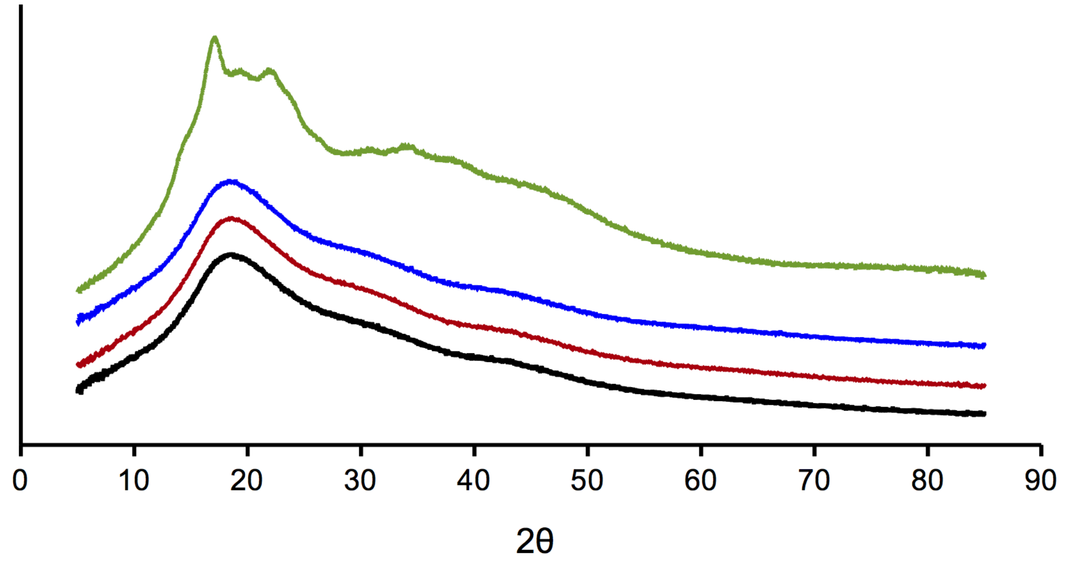

Figure S2. XRPD of the starting material Dx (green) and the polymers **pDx0** (black), **pDx1** (red) and **pDx5** (blue).

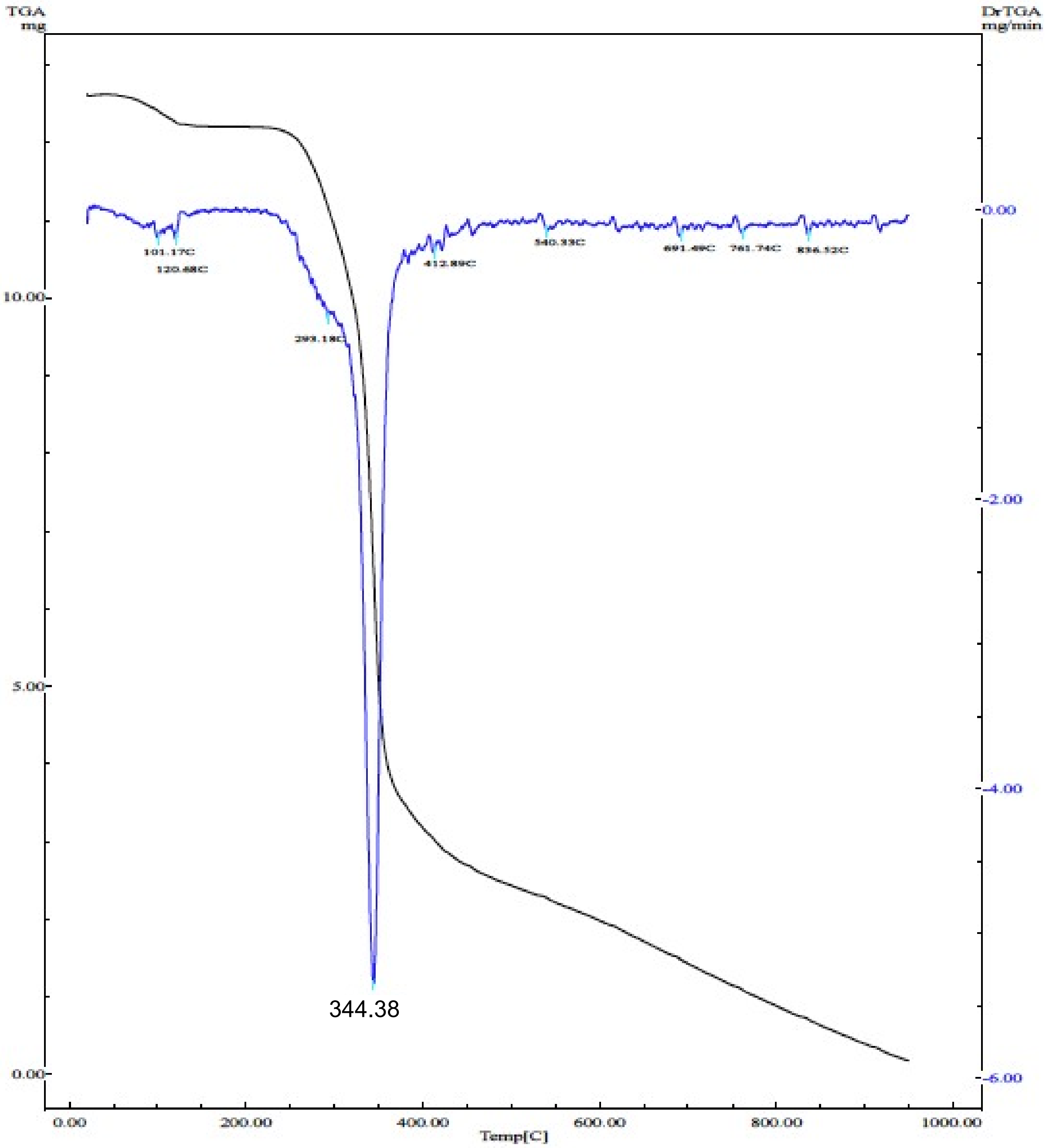

Figure S3. Derivative TGA of **pDx0**. In black is shown the evolution of the mass of the sample as a function of the temperature and in blue the first derivative.

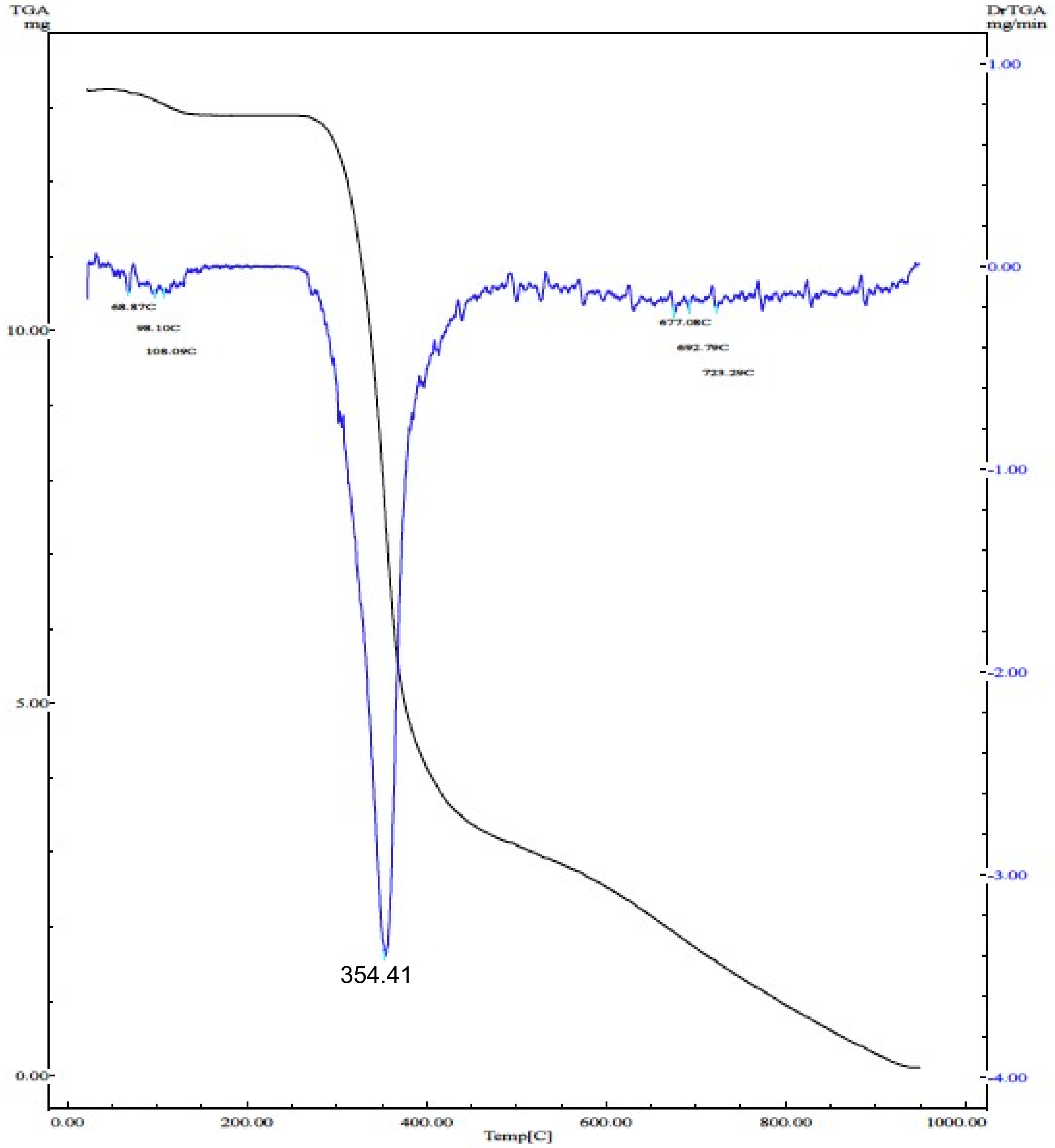

Figure S4. Derivative TGA of **pDx1**. In black is shown the evolution of the mass of the sample as a function of the temperature and in blue the first derivative.

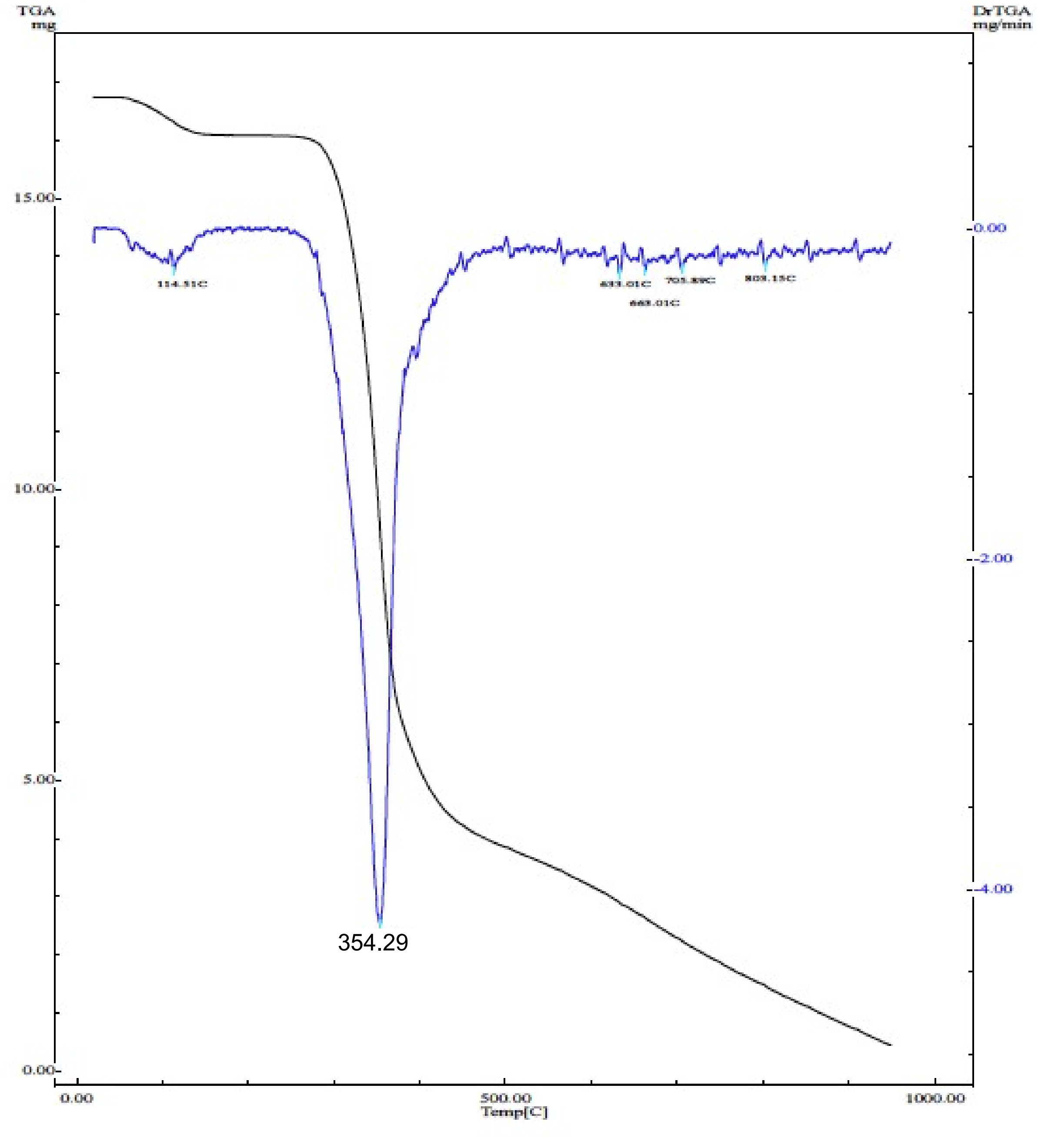

Figure S5. Derivative TGA of **pDx5**. In black is shown the evolution of the mass of the sample as a function of the temperature and in blue the first derivative.

(a)

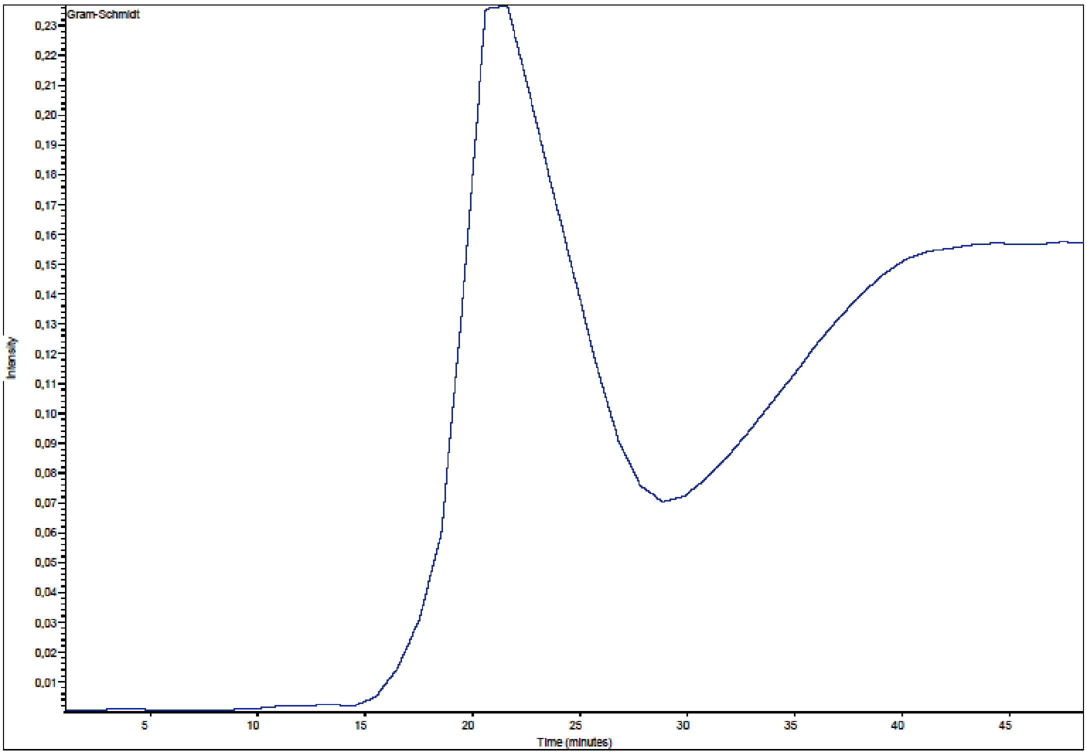

(b)

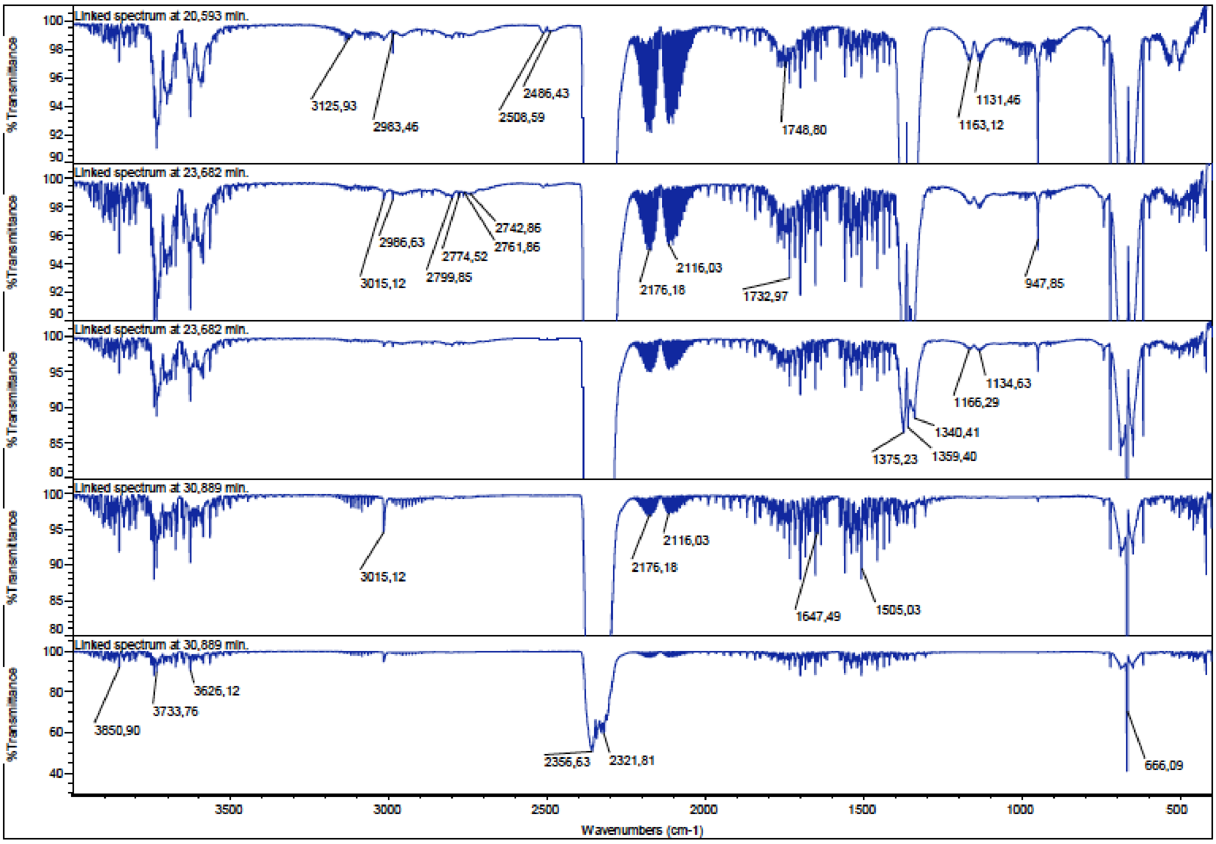

Figure S6. IR−TGA of **pDx0**. (a) Gram−Schmidt. (b) Most representative signals at three single time points 20.6 min, 23.7 min and 34.9 min. The delay between the TG and the FTIR detector is from 4 to 5 min.

(a)

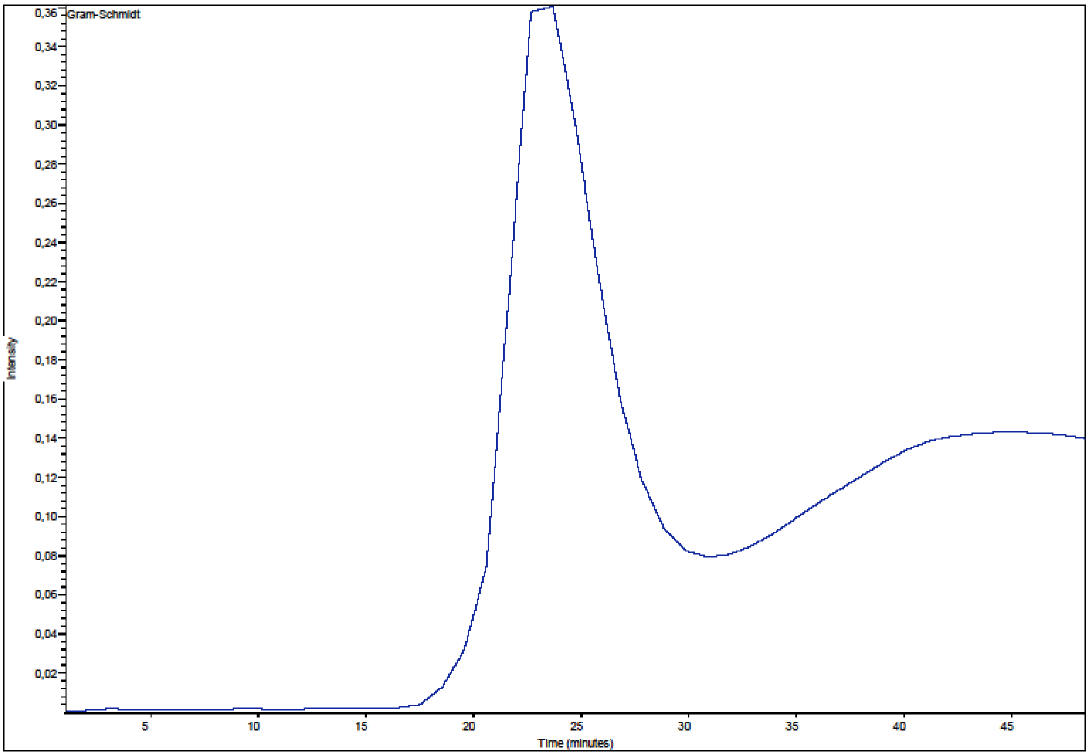

(b)

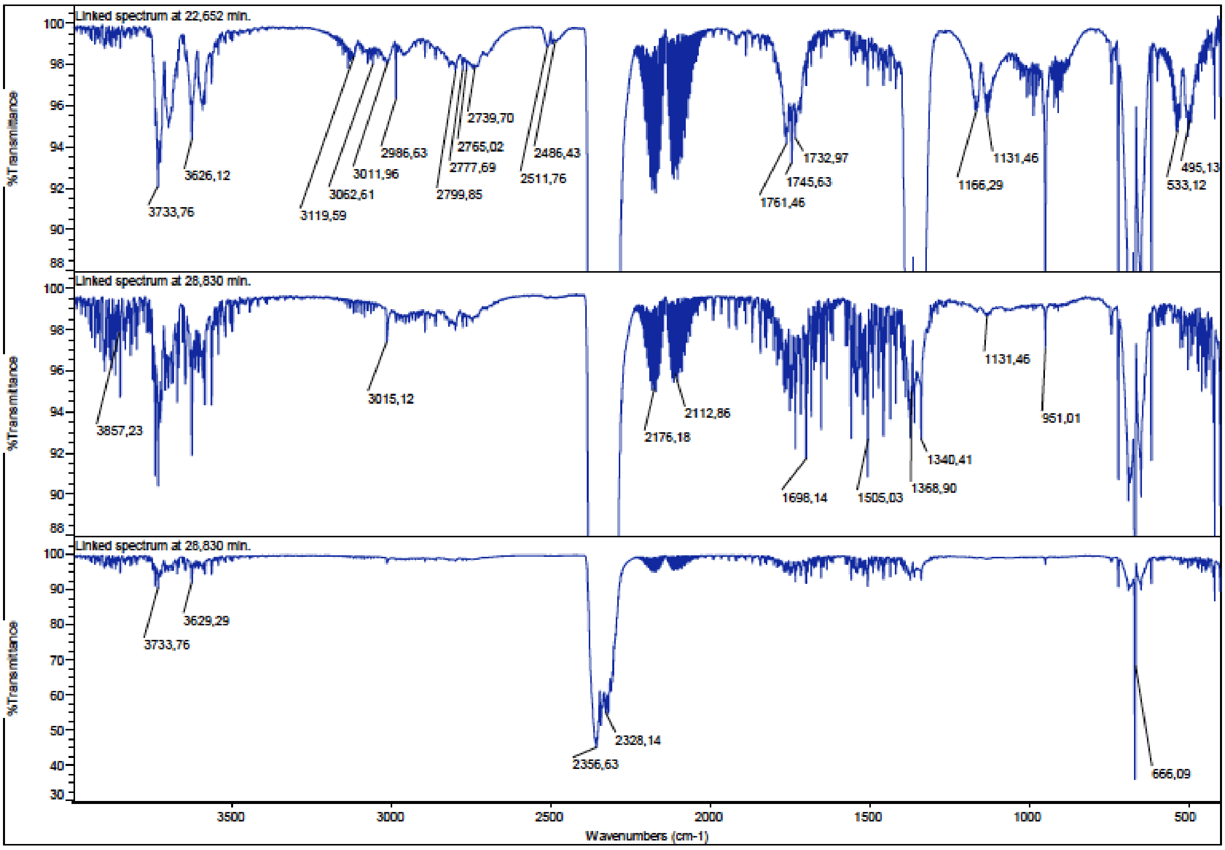

Figure S7. IR−TGA of **pDx1**. (a) Gram−Schmidt. (b) Most representative signals at two single time points 22.6 min and 28.8 min. The delay between the TG and the FTIR detector is from 4 to 5 min.

(a)

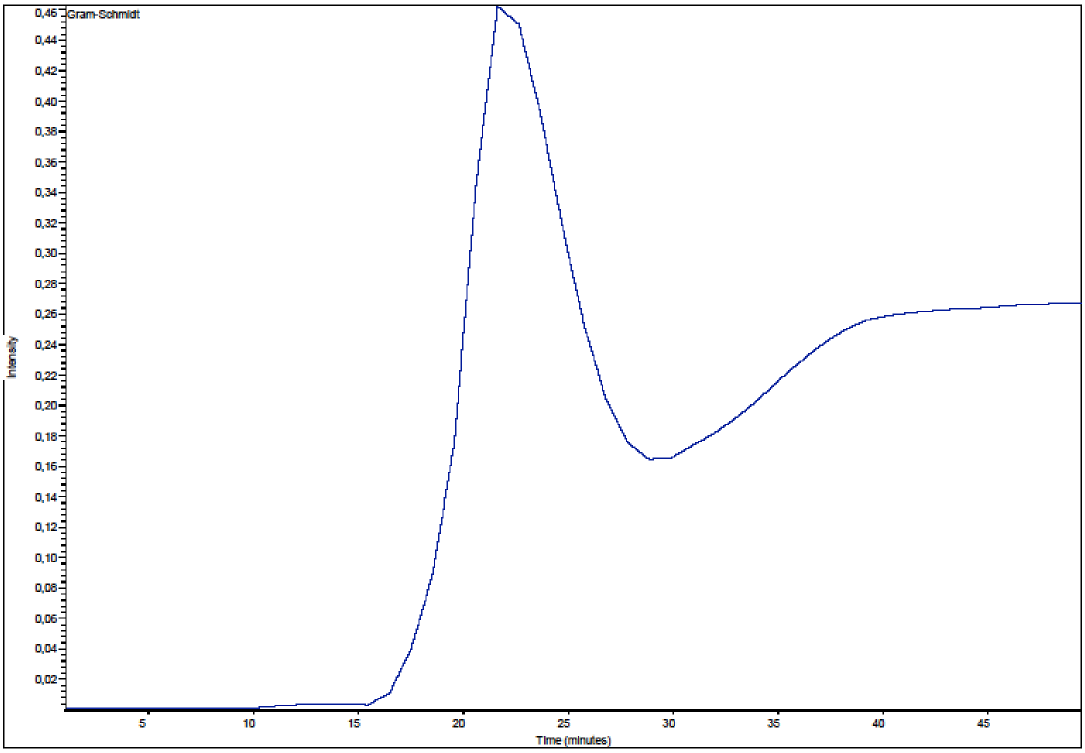

(b)

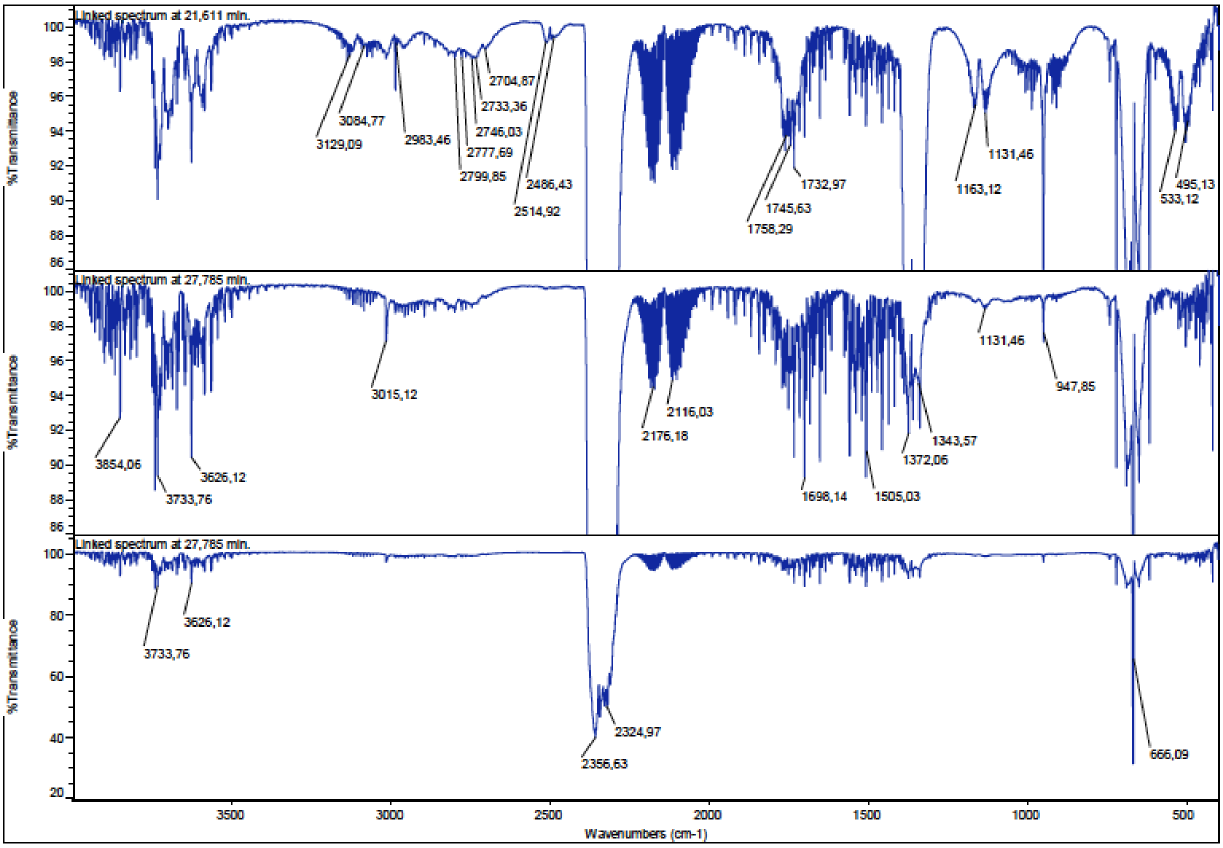

Figure S8. IR−TGA of **pDx5**. (a) Gram−Schmidt. (b) Most representative signals at two single time points 21.6 min and 27.8 min. The delay between the TG and the FTIR detector is from 4 to 5 min.

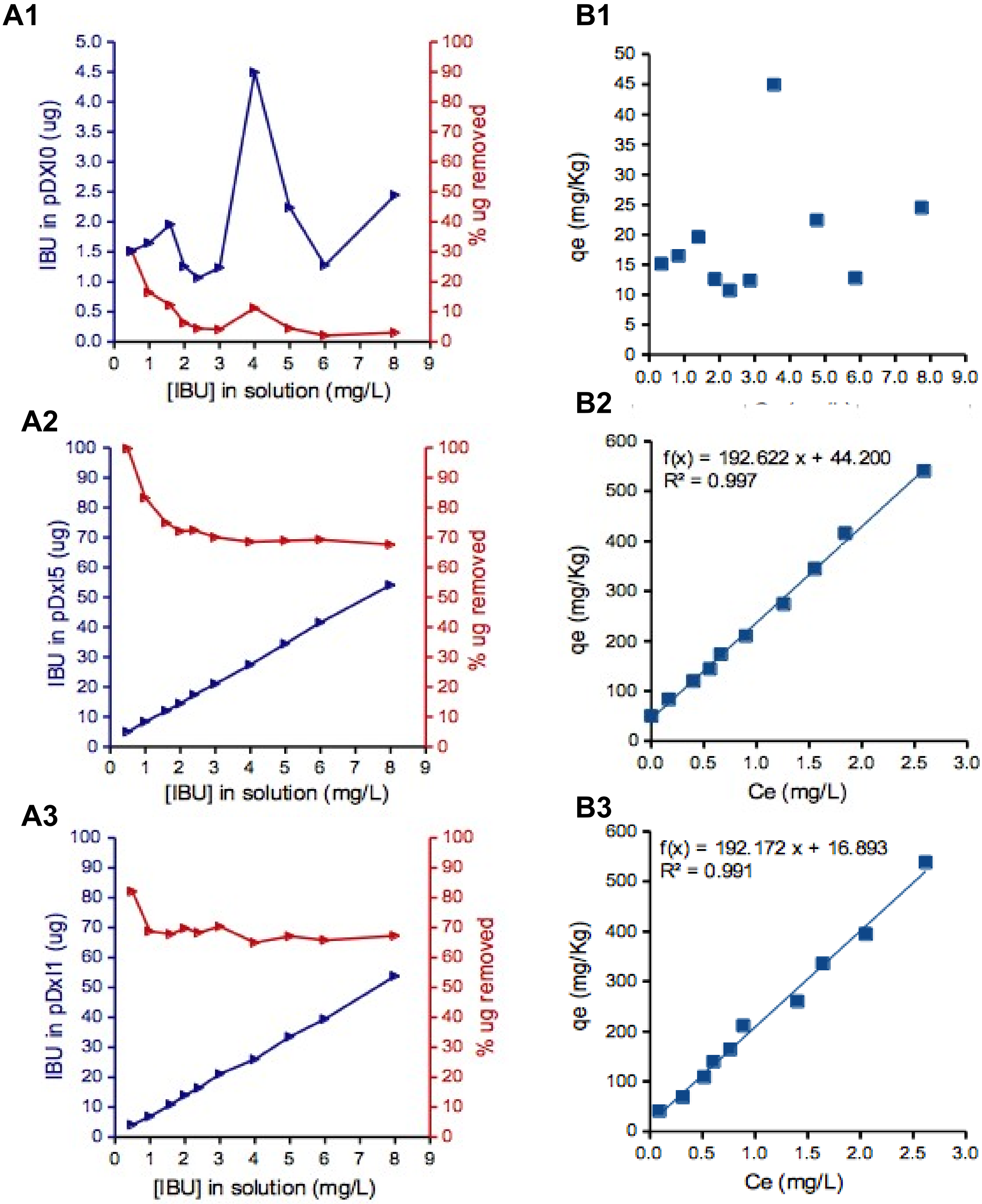

Figure S9. Characterization of the polymers **pDx0** (A1 & B1), **pDx1** (A2 & B2) and **pDx5** (A3 & B3) as scavenger of IBU. A: sorption isotherm showing in blue the amount retained by the polymer and in red the percentage removed from the solution as a function of the initial concentration of IBU. B: estimation of Kd.

Table S1. Mass/volume of reagents used for the synthesis of the polymers

Reagent	Mass (g)	Volume (mL)	Resulting pH
Ibuprofen	0.206 / 1.03		
Water		200	
0.83 M Na_2CO_3		13	9.5
5.5 hour s of gentle stirring			
DVS	5.7	5	
Water		100	
30 min of gentle stirring			
0.83 M Na_2CO_3		187	12
16 hours of gentle stirring			

Table S2. Equations of the isotherm model assayed for the fitting of the experimental sorption data as defined by ISOT_calc[1].

Isotherm (# parameters)	Equation ($q_e = ...$)	Parameter definition[2]
Temkin (2)	$k_1 \cdot \ln\left(C_e\right) + k_2$	k_1 and k_2 are constants of Temkin isotherm and k_1 is related to sorption heat
Freundlich (2)	$K_F \cdot C_e^N$	K_F is adsorption potential<br>N is strength constant associated to heterogeneity (for homogenous systems N=1)
Langmuir (2)	$S_T \dfrac{K_L \cdot C_e}{1 + K_L \cdot C_e}$	S_T is maximum adsorption capacity<br>K_L is affinity constant
Redlich-Peterson (3)	$S_T \dfrac{k \cdot C_e}{\left(1 + C_e\right)^N}$	S_T is maximum adsorption capacity<br>k is related to constants of Redlich-Peterson model<br>N ranges from 0 to 1
Vieth-Sladek (3)	$K_D \cdot C_e + \dfrac{S_T \cdot b \cdot C_e}{1 + b \cdot C_e}$	K_D and b are Vieth-Sladek constants<br>S_T is the maximum adsorption capacity
2-sites Langmuir (4)	$\dfrac{S_{T1} \cdot k_1 \cdot C_e}{1 + k_1 \cdot C_e} + \dfrac{S_{T2} \cdot k_2 \cdot C_e}{1 + k_2 \cdot C_e}$	S_{T1} and $S_{T2}$are the maximum adsorption capacity for sites 1 and 2<br>K_1 and K_2 are affinity constant for sites 1 and 2

1 José L. Beltrán, Joseph J. Pignatello, and Marc Teixidó, "ISOT_Calc: A Versatile Tool for Parameter Estimation in Sorption Isotherms," *Computers & Geosciences*, 2016, 94: 11–17, doi:10.1016/j.cageo.2016.04.008.

2 Mahdieh Mozaffari Majd et al., "Adsorption Isotherm Models: A Comprehensive and Systematic Review (2010–2020)," *Science of The Total Environment*, 2022, 812, 151334, doi:10.1016/j.scitotenv.2021.151334.

Table S3. Elemental analysis of ibuprofen pre-incubated cross−linked polymers. Results are expressed as means ± SD (n = 3).

	**pDx0**	**pDx1**	**pDx5**
**% C**	39.84 ± 0.055	39.85 ± 0.060	39.91 ± 0.330
**% H**	7.35 ± 0.648	7.39 ± 0.142	7.62 ± 0.035
**% S**	6.21 ± 0.428	6.31 ± 0.270	5.85 ± 0.480
